# The effects of symmetrical arrangement on quantum metrology

**DOI:** 10.1038/s41598-017-00544-7

**Published:** 2017-03-24

**Authors:** Yao Jin

**Affiliations:** School of Electronic and Communication Engineering, Guiyang University, Guiyang, Guizhou 550005 China

## Abstract

An obstacle for precision improvement in quantum metrology is the information loss causing by the unavoidable interaction between probe system and environment. Quantum fluctuations are environment no system can be isolated from and it will make the precision of initial parameter estimation of the probe atom decrease with time. After the typical time of the spontaneous decay of the probe atom, the precision is greatly damaged. However, quantum fluctuations can be modified. Our results show that if we put several ancillary atoms beside the probe atom in symmetrical arrangement, the probe atom will be affected by the ancillary atoms indirectly and the information loss of the probe atom causing by the quantum fluctuations will be partially avoided. We find that the retained precision after long time evolution can approaches to $\tfrac{{\bf{9}}}{{\bf{16}}}$ times of the initial precision in condition that the probe atom and three ancillary atoms are located in the vertex of regular tetrahedron.

## Introduction

In estimation theory, parameter can be estimated from probability distribution and the Cramér-Rao bound^1, 2^ tells us there exists uncertainty in parameter estimation. Fisher information is used to describe the precision limit and it has been extended to quantum regime. The so-called quantum Fisher information (QFI) is used to describe how well one can estimate a parameter from a quantum state^1–4^. Since the central task in quantum metrology is to improve the precision of parameter estimation, how to increase the QFI in quantum state becomes a key issue in quantum metrology. Initial correlated systems such as entangled states have been used to increase the QFI in comparison to that in case of using initial uncorrelated systems, thus improve the precision limit of parameter estimation^5–19^. However, in reality, the probe systems are unavoidably in interaction with the environment, the quantum decoherence caused by the environment may decrease the QFI as well as destroy the quantum entanglement in the probe system exploited to improve the precision. In this regard, the dynamics of the estimation precision induced by the interaction between different models of system and environment have been studied^20–39^. As a result, how to control the environmental effects on quantum metrology becomes an important issue.

In quantum sense, quantum fluctuations are environments which no system can be isolated from. The interaction between quantum fluctuations and a probe atom will cause the decoherence behavior of the probe atom. Thus the precision limits of estimating initial atomic parameters decay with time and after the typical time of the spontaneous decay of the probe atom, the precision is greatly damaged^40^. However, quantum fluctuations can be modified. If we put several ancillary atoms beside the probe atom, since all atoms are in interaction with the quantum fluatuations, the probe atom will be affected by the ancillary atoms indirectly and the information loss of the probe atom causing by the quantum fluctuations will also be modified. As the indirect correlations of atoms are determined by the relative arrangement of the atoms, we may wonder what kind of arrangement will help us reduce the information loss and how much will the precision be retained.

## Evolution of total state

We consider the probe system (atom 1) and an ancillary system consisted of *N* − 1 identical two-level ground state atoms interacting with a bath of fluctuating scalar fields in the Minkowski vacuum. We use the natural units and the total Hamiltonian of such a system can be written as

1 $$H={H}_{A}+{H}_{F}+{H}_{I}.$$

Here

2 $${H}_{A}=\sum _{i=1}^{N}\frac{{\omega }_{0}}{2}{\sigma }_{3}^{(i)}$$

is the Hamiltonian of the atoms, where ${\sigma }_{l}^{\mathrm{(1)}}={\sigma }_{l}\otimes {\sigma }_{0}\otimes \ldots \otimes {\sigma }_{0}$, ${\sigma }_{l}^{\mathrm{(2)}}={\sigma }_{0}\otimes {\sigma }_{l}\otimes {\sigma }_{0}\otimes \ldots \otimes {\sigma }_{0}$, …, ${\sigma }_{l}^{(N)}={\sigma }_{0}\otimes \ldots \otimes {\sigma }_{0}\otimes {\sigma }_{l}$, with ${\sigma }_{l}\mathrm{\ (}l=1,2,3)$ being the Pauli matrices and *σ*_0_ being the 2 × 2 unit matrix. We assume all atoms have the same energy level spacing *ω*_0_. *H*_*F*_ denotes the Hamiltonian of the scalar field and the interaction Hamiltonian *H*_*I*_ is taken in analogy to the electric dipole interaction in the weak coupling limit as

3 $${H}_{I}(t)=-\sum _{i=1}^{N}i\mu [{{\rm{\Phi }}}^{(+)}(t,{{\rm{x}}}_{i}){\sigma }_{+}^{(i)}{e}^{i{\omega }_{0}t}-{{\rm{\Phi }}}^{(-)}(t,{{\rm{x}}}_{i}){\sigma }_{-}^{(i)}{e}^{-i{\omega }_{0}t}].$$

Here *μ* denotes the coupling constant. ${{\rm{\Phi }}}^{(+)}(t,{{\rm{x}}}_{i})$ and ${{\rm{\Phi }}}^{(-)}(t,{{\rm{x}}}_{i})$ denote the positive and negative frequency part of the field parameter with ${{\rm{\Phi }}}^{(+)}(t,{{\rm{x}}}_{i})|0\rangle =0$ and $\langle 0|{{\rm{\Phi }}}^{(-)}(t,{{\rm{x}}}_{i})=0$. In interaction picture, the evolution equation of the total state becomes

4 $$i{\partial }_{t}|\psi (t)\rangle ={H}_{I}(t)|\psi (t)\rangle .$$

We assume the parameter to be estimated is the phase factor *ϕ* of the state of the probe system. So the total state of the probe system, ancillary system and environment at initial time can be written as

5 $$|\psi \mathrm{(0)}\rangle =\frac{1}{\sqrt{2}}(|{e}_{1}\rangle +{e}^{i\varphi }|{g}_{1}\rangle )|{g}_{else}\rangle |0\rangle .$$

Here |*e*_1_〉, |*g*_1_〉 denote the excited and ground state of the probe atom. |*g*_*else*_〉 denotes the direct product of vacuum states of all the other atom. We assume the total state at time *t* has the form

6 $$\begin{array}{rcl}|\psi (t)\rangle  & = & \frac{1}{\sqrt{2}}{e}^{i\varphi }|{g}_{1}{g}_{else}\rangle |0\rangle +{b}_{1}(t)|{e}_{1}{g}_{else}\rangle |0\rangle +\sum _{i=2}^{N}{b}_{i}(t)|{g}_{1}{e}_{i}{g}_{else}\rangle |0\rangle \\  &  & +\sum _{\overrightarrow{k}}{b}_{\overrightarrow{k}}(t)|{g}_{1}{g}_{else}\rangle |{1}_{\overrightarrow{k}}\rangle .\end{array}$$

Applying the above equation into the evolution equation of the total state (4), we have

7 $${\dot{b}}_{i}(t)|0\rangle =-\mu \,{{\rm{\Phi }}}^{(+)}(t,{{\rm{x}}}_{i}){e}^{i{\omega }_{0}t}\sum _{\overrightarrow{k}}{b}_{\overrightarrow{k}}(t)|{1}_{\overrightarrow{k}}\rangle ,$$

8 $$\sum _{\overrightarrow{k}}{\dot{b}}_{\overrightarrow{k}}(t)|{1}_{\overrightarrow{k}}\rangle =\sum _{i=1}^{N}\mu \,{{\rm{\Phi }}}^{(-)}(t,{{\rm{x}}}_{i}){e}^{-i{\omega }_{0}t}{b}_{i}(t)|0\rangle .$$

Here [·] denotes the time derivative. In rotating wave approximation, we have

9 $${\dot{b}}_{i}(t)=\sum _{j=1}^{N}{C}_{ij}{b}_{j}(t),$$

where

10 $${C}_{ij}=-{\mu }^{2}{\int }_{0}^{\infty }{e}^{i{\omega }_{0}{\rm{\Delta }}t}\langle 0|{{\rm{\Phi }}}^{(+)}(t,{{\rm{x}}}_{i}){{\rm{\Phi }}}^{(-)}(t^{\prime} ,{{\rm{x}}}_{j})|0\rangle d{\rm{\Delta }}t,$$

with Δ*t* = *t* − *t*′.

## Two atoms case

At first, we let *N* = 2, which means the ancillary system contains only one ground state atom and we use *L* to denote the distance between the two atoms. In this case, the initial state of the total system reduced to

11 $$|\psi \mathrm{(0)}\rangle =\frac{1}{\sqrt{2}}(|{e}_{1}\rangle +{e}^{i\varphi }|{g}_{1}\rangle )|{g}_{2}\rangle |0\rangle .$$

The state at time *t* becomes

12 $$|\psi (t)\rangle =\frac{1}{\sqrt{2}}{e}^{i\varphi }|{g}_{1}{g}_{2}\rangle |0\rangle +{b}_{1}(t)|{e}_{1}{g}_{2}\rangle |0\rangle +{b}_{2}(t)|{g}_{1}{e}_{2}\rangle |0\rangle +\sum _{\overrightarrow{k}}{b}_{\overrightarrow{k}}(t)|{g}_{1}{g}_{2}\rangle |{1}_{\overrightarrow{k}}\rangle .$$

As a result, the evolution equations of *b*_1_(*t*) and *b*_2_(*t*) become

13 $$\begin{array}{rcl}{\dot{b}}_{1}(t) & = & A{b}_{1}(t)+B{b}_{2}(t),\\ {\dot{b}}_{2}(t) & = & B{b}_{1}(t)+A{b}_{2}(t),\end{array}$$

where *A* = *C*_11_ = *C*_22_ and *B* = *C*_12_ = *C*_21_. So we have

14 $$\begin{array}{rcl}{b}_{1}(t) & = & \frac{1}{2\sqrt{2}}[{e}^{(A+B)t}+{e}^{(A-B)t}],\\ {b}_{2}(t) & = & \frac{1}{2\sqrt{2}}[{e}^{(A+B)t}-{e}^{(A-B)t}].\end{array}$$

The coefficients *A* and *B* are determined by the Fourier transformation of the field correlations

15 $$\begin{array}{rcl}{{\mathscr{G}}}_{11}(\lambda ) & = & {\int }_{-\infty }^{+\infty }{e}^{i\lambda ({\rm{\Delta }}t)}\langle 0|{{\rm{\Phi }}}^{(+)}(t,{{\rm{x}}}_{1}){{\rm{\Phi }}}^{(-)}(t^{\prime} ,{{\rm{x}}}_{1})|0\rangle d{\rm{\Delta }}t,\\ {{\mathscr{G}}}_{12}(\lambda ) & = & {\int }_{-\infty }^{+\infty }{e}^{i\lambda ({\rm{\Delta }}t)}\langle 0|{{\rm{\Phi }}}^{(+)}(t,{{\rm{x}}}_{1}){{\rm{\Phi }}}^{(-)}(t^{\prime} ,{{\rm{x}}}_{2})|0\rangle d{\rm{\Delta }}t,\end{array}$$

with

16 $$\begin{array}{rcl}\langle 0|{{\rm{\Phi }}}^{(+)}(t,{{\rm{x}}}_{1}){{\rm{\Phi }}}^{(-)}(t^{\prime} ,{{\rm{x}}}_{1})|0\rangle  & = & -\frac{1}{4{\pi }^{2}}\frac{1}{{({\rm{\Delta }}t-i\epsilon )}^{2}},\\ \langle 0|{{\rm{\Phi }}}^{(+)}(t,{{\rm{x}}}_{1}){{\rm{\Phi }}}^{(-)}(t^{\prime} ,{{\rm{x}}}_{2})|0\rangle  & = & -\frac{1}{4{\pi }^{2}}\frac{1}{{({\rm{\Delta }}t-i\epsilon )}^{2}-{L}^{2}\mathrm{/2}},\end{array}$$

and they can be calculated as

17 $$A=-{\gamma }_{0}\mathrm{/2}-i{\rm{\Omega }}^{\prime} ,$$

with

18 $$\begin{array}{rcl}{\gamma }_{0} & = & {\mu }^{2}{{\mathscr{G}}}_{11}({\omega }_{0})=\frac{{\mu }^{2}{\omega }_{0}}{2\pi },\\ {\rm{\Omega }}^{\prime}  & = & -{\mu }^{2}\frac{P}{2\pi }{\int }_{-\infty }^{\infty }d\lambda \frac{{{\mathscr{G}}}_{11}(\lambda )}{\lambda -{\omega }_{0}},\end{array}$$

and

19 $$B=\frac{{\mu }^{2}}{2\pi i}{\int }_{-\infty }^{\infty }d\lambda \frac{{{\mathscr{G}}}_{12}(\lambda )}{\lambda -{\omega }_{0}-i\epsilon }=-\frac{1}{2}({\gamma }_{12}+iV),$$

with

20 $$\begin{array}{rcl}{\gamma }_{12} & = & {\mu }^{2}{{\mathscr{G}}}_{12}({\omega }_{0})=\frac{{\mu }^{2}\,\sin \,{\omega }_{0}L}{2\pi L},\\ V & = & -\frac{{\mu }^{2}\,\cos \,{\omega }_{0}L}{2\pi L}.\end{array}$$

Then the Bloch vector of the probe atom in Schrodinger picture can be written as

21 $$\begin{array}{rcl}{\omega }_{1}(\tau ) & = & \cos ({\rm{\Omega }}\tau +\varphi )\,\frac{1}{2}[{e}^{-\frac{1}{2}({\gamma }_{0}+{\gamma }_{12}+iV)t}+{e}^{-\frac{1}{2}({\gamma }_{0}-{\gamma }_{12}-iV)t}],\\ {\omega }_{2}(\tau ) & = & \sin ({\rm{\Omega }}\tau +\varphi )\,\frac{1}{2}[{e}^{-\frac{1}{2}({\gamma }_{0}+{\gamma }_{12}+iV)t}+{e}^{-\frac{1}{2}({\gamma }_{0}-{\gamma }_{12}-iV)t}],\end{array}$$

with ${\rm{\Omega }}={\omega }_{0}+{\rm{\Omega }}^{\prime}$. After the study of the evolution of the probe atom, we could come back to our main subject, how the vacuum fluctuations affect the precision of the estimation of initial parameters for a two-level probe atom in the existence of an ancillary two-level atom. When we estimate a parameter *X* from the atomic state *ρ*(*X*), there exists an uncertainty of the parameter *X*, which satisfies the uncertainty conditions^4^

22 $$Var(X)\ge \frac{1}{N{F}_{X}},$$

where *N* represents the repeated times and *F*_*X*_ denotes the quantum Fisher information of parameter *X*, which has the form^38^

23 $${F}_{X}=\{\begin{array}{ll}{|{\partial }_{X}{\boldsymbol{\omega }}|}^{2}+\frac{{({\boldsymbol{\omega }}\cdot {\partial }_{X}{\boldsymbol{\omega }})}^{2}}{1-{|{\boldsymbol{\omega }}|}^{2}}, & |{\boldsymbol{\omega }}| < 1,\\ {|{\partial }_{X}{\boldsymbol{\omega }}|}^{2}, & |{\boldsymbol{\omega }}|=1.\end{array}$$

Applying Eq. (21) into the above equation, we have

24 $${F}_{\varphi }=\frac{1}{4}[{e}^{-({\gamma }_{0}+{\gamma }_{12})t}+{e}^{-({\gamma }_{0}-{\gamma }_{12})t}+2{e}^{-{\gamma }_{0}t}\,\cos (Vt)].$$

We find that the QFI depends on the distance between the two atoms. When ${\omega }_{0}L\ll 1$, *γ*_12_ → *γ*_0_. As a result, due to the existence of the second term, the QFI may partially protected when ${\omega }_{0}L\ll 1$. After long time evolution ($t > {\gamma }_{0}^{-1}$), the QFI remains one quarter of the initial QFI. Let us note here, this condition means that the resonance between two atoms suppress the quantum decay, and keep the information in the system partially. The time scale of validity of this approximation is ${\gamma }_{0}^{-1}$ (*γ*_0_ is the spontaneous decay rate). In reality, the small distance condition can be fulfilled by using long-wavelength molecule. In international system of units, taking LiH whose vibrational transition frequency is *ω*_0_ = 4.21 × 10^13^ Hz as an example, distance of *z* = 1 *μm* satisfies *zω*_0_/*c* = 0.14^41^.

## Three atoms case

Now we let *N* = 3. In three atoms case,

25 $$\begin{array}{rcl}{\dot{b}}_{1}(t) & = & A{b}_{1}(t)+B{b}_{2}(t)+C{b}_{3}(t),\\ {\dot{b}}_{2}(t) & = & B{b}_{1}(t)+A{b}_{2}(t)+D{b}_{3}(t),\\ {\dot{b}}_{3}(t) & = & C{b}_{1}(t)+D{b}_{2}(t)+A{b}_{3}(t),\end{array}$$

where

26 $$\begin{array}{ll}A={C}_{11}={C}_{22}={C}_{33}, & B={C}_{12}={C}_{21},\\ C={C}_{13}={C}_{31}, & D={C}_{23}={C}_{32}.\end{array}$$

From Eq. (25), we have

27 $${\dot{b}}_{1}(t)+m{\dot{b}}_{2}(t)+n{\dot{b}}_{3}(t)=(A+mB+nC)[{b}_{1}(t)+m{b}_{2}(t)+n{b}_{3}(t)],$$

with

28 $$\begin{array}{rcl}B+Dn & = & B{m}^{2}+Cnm,\\ C+Dm & = & Bmn+C{n}^{2}.\end{array}$$

In order to have the protection term in QFI, we need *m* + *n* = −1. As a result, we have

29 $$\{\begin{array}{l}(B-C){n}^{2}+\mathrm{(2}B-C-D)n=0,\\ (B-C){n}^{2}+(B-D)n+(C-D)=0.\end{array}$$

When *B* = *C*, in order to have a solution in Eq. (29), we need *C* = *D*. So we have *B* = *C* = *D*, which means the distances between all of the two atoms are same. In this condition, we have

30 $$\begin{array}{rcl}{b}_{2}(t) & = & {b}_{3}(t),\\ {\dot{b}}_{1}(t) & = & A{b}_{1}(t)+2B{b}_{2}(t),\\ {\dot{b}}_{2}(t) & = & B{b}_{1}(t)+(A+B){b}_{2}(t\mathrm{).}\end{array}$$

Solving the above equations, we have

31 $$\begin{array}{rcl}{b}_{1}(t) & = & \frac{1}{3\sqrt{2}}[{e}^{(A+2B)t}+2{e}^{(A-B)t}],\\ {b}_{2}(t) & = & \frac{1}{3\sqrt{2}}[{e}^{(A+2B)t}-2{e}^{(A-B)t}].\end{array}$$

Then the Bloch vector of the probe atom can be obtained as

32 $$\begin{array}{rcl}{\omega }_{1}(\tau ) & = & \cos ({\rm{\Omega }}\tau +\varphi )\,\frac{1}{3}[{e}^{-\frac{1}{2}({\gamma }_{0}+2{\gamma }_{12}+2iV)t}+2{e}^{\frac{1}{2}({\gamma }_{0}-{\gamma }_{12}+iVt)}],\\ {\omega }_{2}(\tau ) & = & \sin ({\rm{\Omega }}\tau +\varphi )\,\frac{1}{3}[{e}^{-\frac{1}{2}({\gamma }_{0}+2{\gamma }_{12}+2iV)t}+2{e}^{\frac{1}{2}({\gamma }_{0}-{\gamma }_{12}+iVt)}],\end{array}$$

and the QFI becomes

33 $${F}_{\varphi }=\frac{1}{9}[{e}^{-({\gamma }_{0}+2{\gamma }_{12})t}+4{e}^{-({\gamma }_{0}-{\gamma }_{12})t}+4{e}^{-\frac{1}{2}({\gamma }_{0}+2{\gamma }_{12})t}\,\cos \,\mathrm{(3}Vt)].$$

As a result, due to the second part of the above equation, QFI may be partially protected. After long time evolution, the QFI becomes $\tfrac{4}{9}$ times of the initial QFI in condition that ${\omega }_{0}L\ll 1$.

When $B\ne C$, in order to have a solution in Eq. (29), we need *C* = *D* or *B* = *D*. These two cases have the same physical meaning. For *C* = *D*, we have

34 $$\begin{array}{rcl}{b}_{1}(t)-{b}_{2}(t) & = & \frac{1}{\sqrt{2}}{e}^{(A-B)t},\\ {b}_{1}(t)+{b}_{2}(t)+{n}_{1}{b}_{3}(t) & = & \frac{1}{\sqrt{2}}{e}^{(A+B+{n}_{1}C)t},\\ {b}_{1}(t)+{b}_{2}(t)+{n}_{2}{b}_{3}(t) & = & \frac{1}{\sqrt{2}}{e}^{(A+B+{n}_{2}C)t},\end{array}$$

where ${n}_{\mathrm{1,2}}=\frac{-B\pm \sqrt{{B}^{2}+8{C}^{2}}}{2C}$. So we have

35 $${b}_{1}(t)=\frac{1}{2\sqrt{2}}[{e}^{(A-B)t}+\frac{{n}_{2}}{{n}_{2}-{n}_{1}}{e}^{(A+B+{n}_{1}C)t}-\frac{{n}_{1}}{{n}_{2}-{n}_{1}}{e}^{(A+B+{n}_{2}C)t}].$$

As a result, the *e* ^(*A*−*B*)*t*^ part has the same weight with that in two atoms case. So the protection part in QFI is same with that in two atoms case and the retained QFI becomes one quarter of the initial QFI. In conclusion, in three atoms case, the QFI can be partially protected and the largest retained QFI is obtained in condition that the three atoms are in symmetrical arrangement that the distances between all of the two atoms are same.

## N atoms case

Since the largest remained QFI is obtained in the above symmetrical arrangement, now we expand this well arrangement to *N* atoms. We assume the distance of each of the two atoms is same and the distance is small compared to the transition wavelength of the atoms ${\omega }_{0}L\ll 1$. So we have

36 $${C}_{ij}=\{\begin{array}{ll}-{\gamma }_{0}\mathrm{/2}-i{\rm{\Omega }}^{\prime} =A, & i=j,\\ -{\gamma }_{0}\mathrm{/2}-iV\mathrm{/2}\equiv {B}_{0}, & i\ne j.\end{array}$$

As a result,

37 $${b}_{2}(t)={b}_{3}(t)=\cdots ={b}_{N}(t\mathrm{).}$$

So

38 $$\begin{array}{rcl}{\dot{b}}_{1}(t) & = & A{b}_{1}(t)+{B}_{0}(n-\mathrm{1)}{b}_{2}(t),\\ {\dot{b}}_{2}(t) & = & {B}_{0}{b}_{1}(t)+[A+{B}_{0}(n-\mathrm{2)]}{b}_{2}(t\mathrm{).}\end{array}$$

Solving the above equations, we have

39 $$\begin{array}{rcl}{b}_{1}(t) & = & \frac{1}{\sqrt{2}N}[{e}^{[A+(N-\mathrm{1)}{B}_{0}]t}+(N-\mathrm{1)}{e}^{(A-{B}_{0})t}],\\ {b}_{2}(t) & = & \frac{1}{\sqrt{2}N}[{e}^{[A+(N-\mathrm{1)}{B}_{0}]t}-(N-\mathrm{1)}{e}^{(A-{B}_{0})t}].\end{array}$$

The Bloch vector of the probe atom is obtained as

40 $$\begin{array}{rcl}{\omega }_{1}(\tau ) & = & \cos ({\rm{\Omega }}\tau +\varphi )\,\frac{1}{N}[{e}^{-\frac{1}{2}[N{\gamma }_{0}+i(N-\mathrm{1)}V]t}+(N-\mathrm{1)}{e}^{\frac{1}{2}iVt}],\\ {\omega }_{2}(\tau ) & = & \sin ({\rm{\Omega }}\tau +\varphi )\,\frac{1}{N}[{e}^{-\frac{1}{2}[N{\gamma }_{0}+i(N-\mathrm{1)}V]t}+(N-\mathrm{1)}{e}^{\frac{1}{2}iVt}].\end{array}$$

So the QFI can be calculated as

41 $${F}_{\varphi }=\frac{1}{{N}^{2}}[{e}^{-N{\gamma }_{0}t}+{(N-\mathrm{1)}}^{2}+\mathrm{2(}N-\mathrm{1)}{e}^{-\frac{1}{2}N{\gamma }_{0}t}\,\cos (NVt)].$$

**Figure 1:**
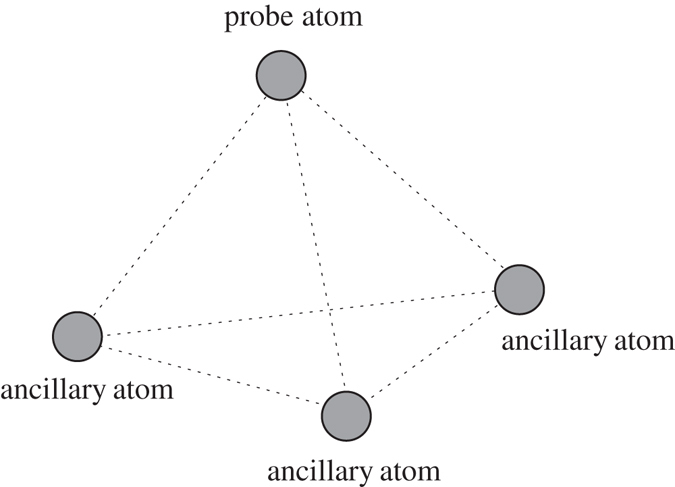
The probe atom and the three ancillary atoms located in the vertex of regular tetrahedron.

Due to the second part of the above equation, the retained QFI becomes ${(\tfrac{N-1}{N})}^{2}$ times of the initial QFI. However, in fact, the symmetric arrangement condition can be fulfilled when *N* = 4 at most. In this case, the probe atom and the three ancillary atoms are located in the vertex of regular tetrahedron as is shown in Fig. (1). The retained QFI then becomes $\tfrac{9}{16}$ times of the initial QFI.

## Conclusion

In conclusion, we have studied the dynamics of QFI of parameters of initial state of a static two-level probe atom in the Minkowski vacuum in the existence of *N* − 1 two-level ancillary atoms. Our results show that the QFI, thus the precision limit of the estimation of probe atom will be retained after long time evolution with proper arrangement of the atoms. The largest retained QFI is obtained in symmetrical arrangement that the distances between all of the two atoms are same and the retained precision approaches to $\tfrac{9}{16}$ times of the initial precision for *N* = 4 at most.
